# Outbreak of Oleander (*Nerium oleander*) Poisoning in Dairy Cattle: Clinical and Food Safety Implications

**DOI:** 10.3390/toxins12080471

**Published:** 2020-07-24

**Authors:** Luigi Ceci, Flavia Girolami, Maria Teresa Capucchio, Elena Colombino, Carlo Nebbia, Fabio Gosetti, Emilio Marengo, Fabrizio Iarussi, Grazia Carelli

**Affiliations:** 1 Department of Veterinary Medicine, University of Bari, 70121 Bari, Italy; luigi.ceci@uniba.it (L.C.); grazia.carelli@uniba.it (G.C.); 2 Department of Veterinary Sciences, University of Torino, 10124 Torino, Italy; mariateresa.capucchio@unito.it (M.T.C.); elena.colombino@edu.unito.it (E.C.); carlo.nebbia@unito.it (C.N.); 3 Department of Earth and Environmental Sciences, University of Milano-Bicocca, 20126 Milano, Italy; fabio.gosetti@unimib.it; 4 Department of Sciences and Technological Innovation, University of Eastern Piedmont, 13100 Vercelli, Italy; emilio.marengo@uniupo.it; 5 Department of Emergency and Organ Transplantation, University of Bari, 70124 Bari, Italy; fabrizio.iarussi@uniba.it

**Keywords:** bovine, toxicosis, dairy products, public health, risk

## Abstract

Oleander is a spontaneous shrub widely occurring in Mediterranean regions. Poisoning is sporadically reported in livestock, mainly due to the ingestion of leaves containing toxic cardiac glycosides (primarily oleandrin). In this study, 50 lactating Fleckvieh cows were affected after being offered a diet containing dry oleander pruning wastes accidentally mixed with fodder. Clinical examination, electrocardiogram, and blood sampling were conducted. Dead animals were necropsied, and heart, liver, kidney, spleen, and intestine were submitted to histological investigation. Oleandrin detection was performed through ultra-high performance liquid chromatography-tandem mass spectrometry in blood, serum, liver, heart, milk, and cheese samples. Severe depression, anorexia, ruminal atony, diarrhea, serous nasal discharge, tachycardia, and irregular heartbeat were the most common clinical signs. The first animal died within 48 h, and a total of 13 cows died in 4 days. Disseminated hyperemia and hemorrhages, multifocal coagulative necrosis of the cardiac muscle fibers, and severe and diffuse enteritis were suggestive of oleander poisoning. The diagnosis was confirmed by the presence of oleandrin in serum, liver, heart, milk, and cheese. Our results confirm the high toxicity of oleander in cattle and report for the first time the transfer into milk and dairy products, suggesting a potential risk for the consumers.

## 1. Introduction

Oleander is an evergreen plant, widespread all over the world, belonging to the Apocynaceae family, whose most prevalent species are *Nerium oleander* (common oleander) from the Mediterranean basin and Asia [1] and *Thevetia peruviana* (yellow oleander) from tropical America [2]. Its toxicity has been known since ancient times: in India, before Christ, the shrub was called Kajamaraka, “the herb that makes the horse die” [3]. Oleander poisoning may be fatal for humans, animals, and even for some insects [4]. Indeed, all parts of the plant are highly toxic as they contain several non-digitalis cardiac glycosides, including oleandrin, nerin, digitoxigenin, and olinerin, collectively referred to as cardenolides. Due to a relatively high lipophilicity resulting in a rapid and extensive gastrointestinal absorption and a slow urinary excretion rate, the most active molecule is oleandrin [5]. The mechanism of toxicity acts through the inhibition of the Na^+^-K^+^-ATPase pump in cardiomyocytes, increasing the intracellular Na^+^ concentration. This affects the Na^+^/Ca^2+^ exchange channels, leading to the intracellular increase of Ca^2+^ levels that determines a positive inotropic effect, as well as a rise of the resting membrane potential with consequent augmented excitability and automaticity of myocardial cells. An interference with the vagal tone leading to slow atrioventricular conduction and ventricular arrhythmias has been also documented [6]. Furthermore, the extracellular excretion of K^+^ induces hyperkalemia that contributes to exacerbating the onset of arrhythmias and hypotension.

In humans, mortality due to oleander usually involves the voluntary ingestion of decoctions or portions of the vegetable for suicidal purposes [7]. However, accidental poisonings have been described, particularly in children, where one leaf can be lethal [4,6]. Accidental poisonings have been frequently described in different animal species [8,9,10], including cattle [11,12,13,14,15]. Although oleander is unpalatable due to a bitter and pungent taste [16], grazing animals may ingest parts of the shrub in arid areas or during the dry season when there is scarcity of fodder. However, poisoning can also be attributed to human managerial errors when oleander is unintentionally mowed, crushed, and mixed with feed. Animals can also be intoxicated after the ingestion of water containing fallen and macerated leaves. Fortunately, the risk of poisoning is reduced by the presence of saponins, which in monogastrics may induce vomiting and facilitate the elimination of the ingested toxic vegetables [17]. The lethal dose of dried *Nerium oleander* leaves (LD) varies according to the animal species. It seems that bovines are more sensitive compared to small ruminants, the LD being 50 mg/kg for cattle, 110 mg/kg for goats, and 250 mg/kg for sheep [1,18,19].

In the present study, the authors describe the occurrence of a cattle herd intoxication in Southern Italy due to the accidental presence of oleander leaves in the hay. The diagnosis was performed through clinical, histopathological, and chemical investigations. The presence of oleandrin in milk and dairy products was also examined.

## 2. Results

The poisoning episode occurred in a dairy farm located in the countryside of Cisternino (Brindisi, Italy), where 50 Fleckvieh lactating cows were affected. At day 0, the breeder noticed that the lactating animals displayed discomfort with depression, anorexia, lack of rumination, and hypogalaxia. Suspecting a possible feed poisoning, the breeder promptly removed the feed ration and called the farm veterinarian. At day 1, some cows worsened, showing diarrhea, severe depression, and prolonged sternal decubitus. Thus, a symptomatic therapy with rehydrating fluids and atropine was performed. However, a cow died late in the evening and 12 other animals died on days 2 and 3, showing pedaling, convulsive movements, increased frequency of bellowing, and coma. The farm veterinarian informed the official veterinarians of the local health service who decided to discard the farm milk for 15 days as a precautionary measure. The Internal Medicine Unit of the Dept. of Veterinary Medicine, University of Bari, was asked for a consultation. A general examination of the herd was performed. Further, complete clinical examination, electrocardiogram (ECG), and blood sampling for a hematobiochemical profile were carried out on four animals showing the most severe symptoms.

**Figure 1:**
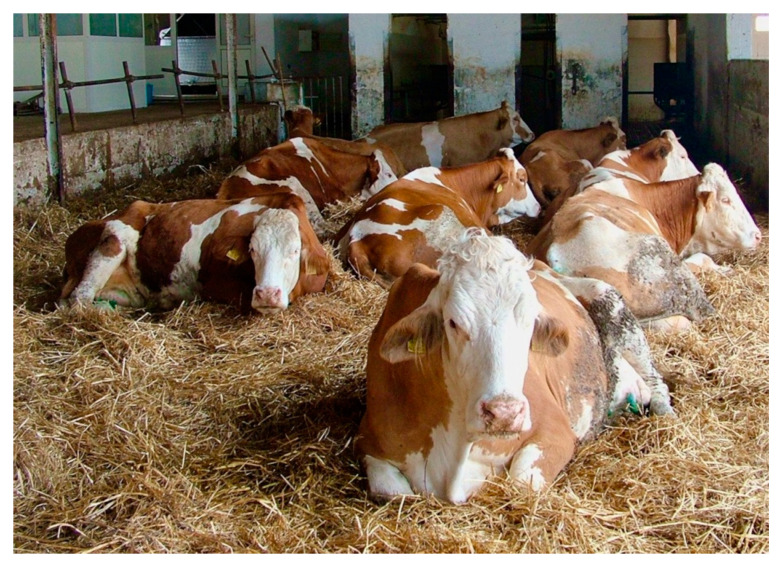
Poisoned cows showing depression and general weakness.

At the herd general clinical examination, almost all animals showed, as a predominant sign, varying degrees of depression and weakness; many subjects were in sternal decubitus with lowered ears, and some were soporous (Figure 1).

**Figure 2:**
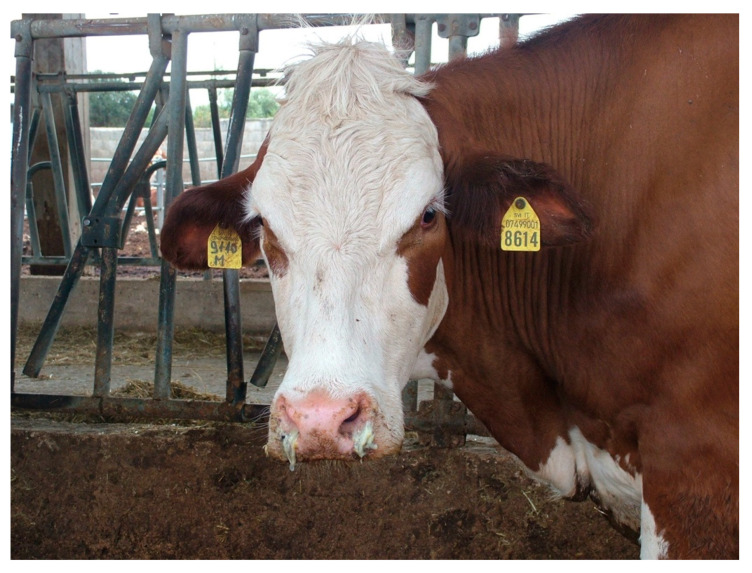
One of the affected cows showing a yellowish mucous-fibrinous nasal discharge.

The other observable symptoms were highly variable in the different subjects. Some cows showed evident nasal and lacrimal discharge with dense yellowish mucous-fibrinous exudate (Figure 2), while others were mildly dehydrated, with dry mucous membranes and muzzle, and slightly sunken eyes.

**Figure 3:**
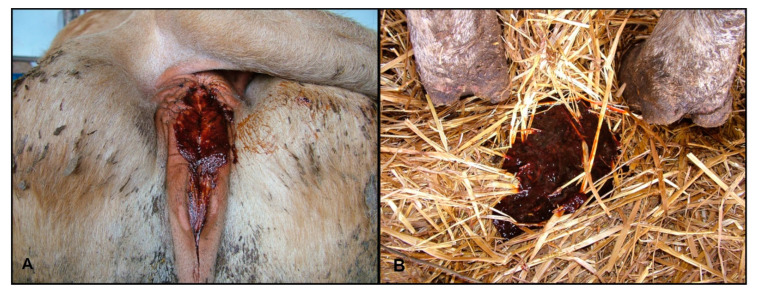
(**A**) Perineal area of a diseased cow with traces of hemorrhagic diarrhea; (**B**) Hemorrhagic diarrhea.

Several animals showed ptyalism. In some cases, the rumen was atonic with mild meteorism, and most of the animals showed lack of rumination. There were varying degrees of abdominal pain (colic) and false kyphosis. Diarrhea was present in most of the animals with different characteristics: aqueous, translucent liquid with fibrin and rusty blood traces, and pasty stools with blood streaks. In some cattle, the perianal area was smeared with hemorrhagic stools (Figure 3A,B).

**Figure 4:**
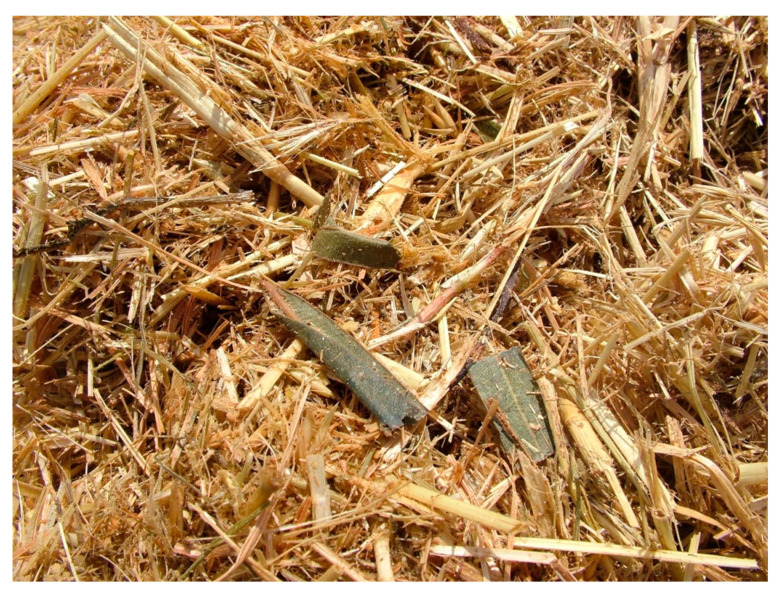
Oleander leaves mixed into the feed ration.

Some animals showed tachypnea, cough, and mainly abdominal discordant breathing. Other cows displayed pollakiuria, bruxism, and muscular fibrillations. The body temperature was always normal. A careful check of the remaining feed ration that was removed by the farmer revealed the presence of intact and fragmented oleander leaves mixed into the hay (Figure 4).

At the clinical examination of the four animals with the most severe symptoms, depression (drowsiness, dizziness, prolonged sternal decubitus, slow and staggering gait), sero-fibrinous nasal and lacrimal discharge, slight colic pain, ruminal atony, and watery diarrhea with blood streaks were present. Heart auscultation showed a splitting of the first tone in two animals, and different arrhythmias in the other two. At auscultation of lungs, strengthened vesicular murmur and slight rattles were detected. The body temperature remained normal.

The complete blood count (CBC) of the four examined cows was within the normal range, while several biochemical parameters were altered. Alkaline phosphatase (ALP), lactate dehydrogenase (LDH), creatine kinase (CK), total protein, urea, and creatinine were dramatically increased, with mean values equal to 359.8 IU/L (reference interval (RI) 29–99 IU/L), 2216.5 IU/L (RI 692–1445 IU/L), 321.5 IU/L (RI 4.8–12.1 IU/L), 8.9 g/dL (RI 5.9–7.7 g/dL), 184.8 mg/dL (RI 15–35 mg/dL), and 3.4 mg/dL (RI 0.70–1.10 mg/dL), respectively. As regards ions, calcium was slightly diminished (7.9 mg/dL, RI 8.4–10.5 mg/dL), while chloride (298 mEq/L, RI 90–105 mEq/L) and magnesium (2.8 mg/dL, RI 1.7–2.2 mg/dL) were augmented.

**Figure 5:**
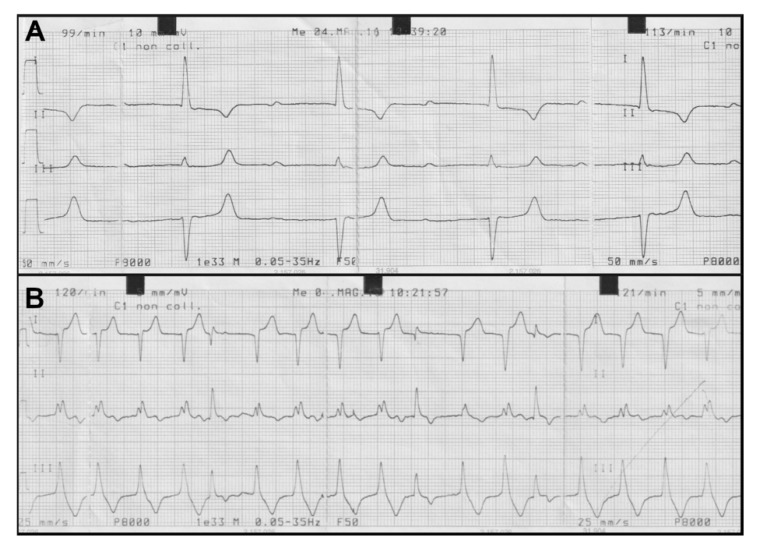
Electrocardiogram (ECG) traces showing two representative observed arrhythmias. (**A**) Atrioventricular block of 1st grade frequency with variable tachycardia from 99 to 113 beats/minute; (**B**) Paroxysmal ventricular tachycardia with severe ventricular arrhythmias with a frequency of 122 beats/minute.

Abnormalities of the cardiac rhythm detected at the heart auscultation were confirmed at the ECG examination. In particular, we observed an atrioventricular block of 1st degree with a variable frequency from 99 to 113 beats/minute (Figure 5A) and a paroxysmal ventricular tachycardia with ventricular arrhythmias with a frequency of 122 beats/minute (Figure 5B).

The necropsy performed on all the dead animals showed a diffuse congestion of visceral organs including liver, kidneys, lungs, abomasum, and intestine. Multifocal, mild hemorrhages in the ventricular endocardium were also observed. The lungs were edematous, with frothy contents in the bronchi. Mild hydrothorax, hydropericardium, and ascites were also present.

**Figure 6:**
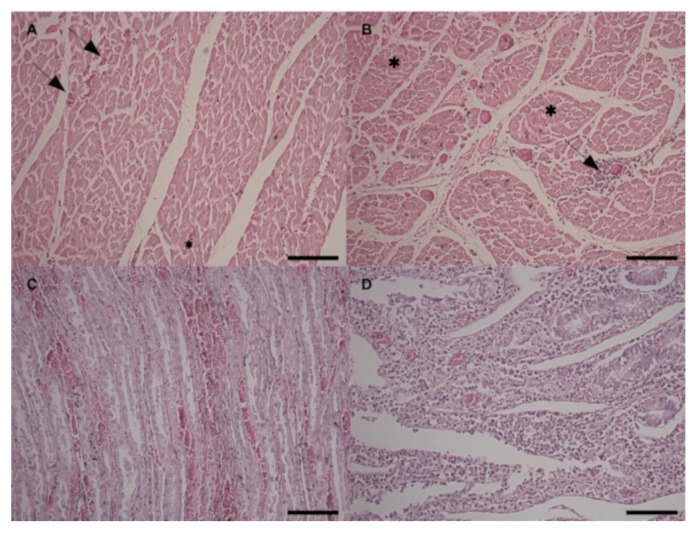
Histopathological findings in the Haematoxylin and Eosin staining at 20×. (**A**) Heart, right papillary muscle area. Mild multifocal hyperemia (black asterisks) and hemorrhages (black arrows); (**B**) Heart, right papillary muscle area. Multifocal to disseminated degenerated fibers (black asterisks), mild, multifocal non suppurative interstitial myocarditis (black arrows); (**C**) Kidney. Mild, multifocal hyperemia and hemorrhages; multifocal tubular degeneration; (**D**) Duodenum. Severe diffuse chronic enteritis characterized by hyperemia of the mucosa and lymphoplasmacytic/eosinophilic infiltration.

At the histological examination, all the cardiac areas showed mild multifocal hyperemia and hemorrhages (Figure 6A) with mild multifocal non suppurative interstitial inflammatory infiltrates (Figure 6B). Mild to severe multifocal arteriosclerosis of small intramural coronary arteries and extramural coronary arteries was also observed. Moreover, the left and right papillary muscles and the left and right ventricular wall areas showed disseminated hypotrophic hyper-eosinophilic shrunken muscular fibers representing different stages of necrosis (Figure 6B). Mitral and tricuspid valves showed mild endocardiosis. Kidneys showed diffuse passive hyperemia and mild, multifocal hemorrhages, especially in the renal medulla. Mild, multifocal lymphoplasmacytic interstitial nephritis and multifocal tubular degeneration were also detected (Figure 6C). In the liver, perilobular non suppurative acute hepatitis with moderate diffuse hyperemia was observed. Spleen showed moderate diffuse depletion of the red and white pulp. Regarding the gastrointestinal tract, the esophagus showed chronic active inflammation of the mucosa. Small intestine presented severe diffuse chronic enteritis characterized by hyperemia of the mucosa and lymphoplasmacytic/eosinophilic infiltration invading the mucosa and partially submucosa (Figure 6D).

Multifocal gastrointestinal associated lymphoid tissue activation was also observed. Colon and cecum showed similar findings with a greater number of eosinophils and a moderate multifocal hyperemia also in the submucosa. Meseraic (mesenteric) lymph nodes presented severe and diffuse reactive hyperplasia associated with rarefaction of the lymphatic centers, interfollicular zone, and medullary cords with disseminated macrophages containing brownish pigment.

The hypothesis of oleander poisoning was confirmed by the chemical analyses performed by ultra-high performance liquid chromatography-tandem mass spectrometry (UHPLC-MS/MS) on blood, serum, heart, and liver samples from three poisoned cows. Indeed, oleandrin concentration in blood and serum was around 1 ng/mL in all animals, while more than 10-fold higher levels were detected in heart (15.29 ± 0.79 ng/g) and liver (15.94 ± 0.44 ng/g). Oleandrin concentrations similar to those in blood and serum were measured also in milk (1.25 ± 0.99 ng/mL) and cheese (0.82 ± 0.02 ng/g).

A symptomatic therapy to reduce cardiac abnormalities and to restore the hydro-saline balance was conducted as suggested in the literature [20]. After six days from the removal of the poisoned feed, and after four days of symptomatic therapy, the remaining 37 lactating cows came to a complete recovery and went to pasture.

## 3. Discussion

The study thoroughly described the symptoms and the clinical and pathological findings of oleander intoxication in dairy cows. In addition, we highlighted the need of an accurate diagnosis through chemical analysis and explored the possibility that oleandrin could be transferred to animal products (i.e., milk and cheese).

The clinical picture here described is in agreement with what has been reported in the literature [11,13]. Oleander poisoning is characterized by polymorphous symptoms, whose onset and severity vary according to the amount of active principles ingested [11]. Indeed, we observed a rapid onset of symptoms within the first 24 h after the ingestion of the plant, followed by the death of the first animal within 48 h. The other 12 animals died within the following four days. The main clinical signs are related to disorders of the cardiac, gastrointestinal, and nervous systems [12,13]. Accordingly, the animals showed depression, sero-fibrinous nasal and ocular discharge, slight colic pain, ruminal atony, and diarrhea. It should be noted that the clinical presentation varied among individual members of the herd regarding the type and severity of toxicity. We believe this may be related to variation in the dose of the ingested poison.

The gastrointestinal tract involvement in ruminants results frequently in abdominal pain, atony and tympanism; however, diarrhea has been observed in acute accidental oleander poisoning in cattle as well as in other animal species [11,21]. It is suggested that such clinical signs are probably due to the direct contact of the oleander toxins with the mucosa rather than being secondary to nervous or circulatory involvement [21] as reported for humans [22]. Similarly, the neurological disorders are caused by the direct effect of the toxins, which are able to cross the blood–brain barrier. However, vascular endothelial damage and acute heart failure are likely to contribute to the central nervous system impairment [19].

As oleander contains cardiac glycosides, the heart is the most affected organ. Thus, a meticulous heart auscultation along with an ECG examination is essential for the diagnosis [19]. The atrioventricular block of 1st degree displayed by two of the examined cows has been attributed by several authors to the effect of oleandrin on the conduction tissue, even if such an anomaly can also be found in healthy animals [23]. Indeed, the inhibition of the Na^+^-K^+^-ATPase pump in cardiomyocytes also stimulates the sympathetic outflow that sensitizes the myocardium and exaggerates all the toxic effects. Such a phenomenon determines a reduction in the normal electrical conductivity of the myocardium that leads to conduction blocks and ventricular arrhythmias [19]. Conversely, the other detected alterations of the cardiac rhythm (i.e., premature ventricular complexes and paroxysmal ventricular tachycardia with S-T segment slanting) could originate from coronary ischemia and may be diagnostically indicative of oleander poisoning within a generalized toxicosis. Indeed, it has been reported that in experimentally intoxicated goats, the sinus bradycardia and the 2nd degree atrioventricular block usually occur in the first hours after oleander administration [19]. Subsequently, the excitatory forms prevail (i.e., sinus tachycardia, ventricular extrasystolia, ventricular tachycardia, ventricular fibrillation), as also reported for experimentally poisoned sheep [19,21]. Thus, we can assume that in our case the death of the animals occurred following ventricular tachycardia and fibrillation.

Common blood chemistry changes associated with oleander intoxication in humans include increased azotemia, hyperglycemia, elevated CK and LDH, and hyperkalemia; furthermore, persistent hyperglycemia and hyperkalemia are considered poor prognostic indicators of cardiac glycoside toxicosis [24]. Accordingly, in the present study the results of the hematobiochemical analyses were suggestive of kidney failure, liver damage, and a diffuse inflammatory status, involving mostly the cardiac and the skeletal muscle tissues. Such findings were in agreement with those reported in experimentally poisoned sheep [18]. Apart from a slight electrolyte imbalance, neither hyperglycemia nor hyperkalemia were noticed. Nevertheless, the prognostic value of such alterations in veterinary patients has not been determined, and hypoglycemia, rather than hyperglycemia, has been associated with oleander toxicity in a dog [25].

Taken together, the clinical findings associated with the detection of oleander leaves in the hay pointed to the suspicion of oleander poisoning. This hypothesis was further corroborated by the macroscopic and histopathological findings. Indeed, congestion of kidney, liver, lungs, and intestine and hemorrhages of the ventricular endocardium, hydrothorax, hydropericardium, and ascites have also been reported in sheep and goats experimentally poisoned by *Nerium oleander* [21,26]. Histologically, the main lesions observed in the present study were myocardial degeneration and necrosis, non-suppurative nephritis, hepatitis and enteritis, with congestion and hemorrhages in all the examined organs. The same histological lesions have been observed in experimentally intoxicated sheep, goats, and mice [21,26,27]. Such lesions could be attributed to the toxic action of oleander cardenolides that impair the cardiac and renal function through the inhibition of the Na^+^-K^+^-ATPase pump [28,29]. It is likely that the development of cardiac arrhythmias is related also to the histological alterations of the myocardium. Indeed, the increase of cytosolic Ca^2+^, subsequent to the block of the pump, provokes the activation of phospholipases and proteases that in turn cause phospholipid degradation and cytoskeletal damage, respectively [30]. Moreover, the nephritis and the tubular degeneration here detected were responsible for the renal failure depicted by the increased serum levels of urea and creatinine, as described in oleander poisoned goats [26]. The pathogenetic mechanism of cardiac glycosides on tissues other than heart has not been completely elucidated. However, toxic glycosides may induce the release of pro-inflammatory cytokines, such as TNF-α, and stimulate the triggering of inflammatory mediators, such as IL-1, IL-6, TGF-β, and prostaglandins, that facilitate inflammatory cellular infiltration and degeneration in heart, kidney, liver, and gastrointestinal organs [27].

Currently available techniques for the detection of *Nerium oleander* toxic components include digoxin fluorescence polarization immunoassay, based on the molecular similarities between digoxin and oleandrin, thin-layer chromatography, high-performance liquid chromatography of the fluorescent derivative, and LC-MS/MS [31]. However, most analytical methods are not sufficiently sensitive or specific to detect and quantify oleandrin in biological fluids and tissues. The most recommended method that provides a rapid and unequivocal determination is LC-MS/MS [32]. Accordingly, the definitive diagnosis of oleander intoxication in our study was performed through a highly sensitive UHPLC-MS/MS method recently developed and validated for oleandrin determination in bovine samples [33]. The detection of elevated concentrations of the toxic principle in heart and liver is consistent with the clinical and pathological findings, underlining the main role of these two organs in the poisoning pathogenesis. Finally, it should be underlined that the diagnosis of oleander intoxication derives from the combination of clinical/pathological and analytical findings. In the absence of an accurate clinical examination, the detection of plant parts (e.g., leaves) or toxic components in the rumen is not sufficient [15].

To the authors’ best knowledge, there are no published data about the presence of oleandrin into the milk or cheese from poisoned animals. Thus, this is the first report that sets up and validates a specific analytical method for such matrices of animal origin, and documents the transfer of oleandrin in dairy milk and derived products, with a potential consumer health concern. Even if human mortality associated with oleander ingestion is generally low [4], except for suicide attempts, infants and children are particularly sensitive, and even very low concentrations can be life threatening. Taking into account that they represent the largest consumers of milk, and that the concentrations we measured in milk and cheese were similar to those detected in cow blood and serum, a better evaluation of the risk of toxicosis related to the consumption of milk from poisoned animals is advisable. In this respect, a limitation of our study is that a time-course milk sampling and analysis is missing. It would be useful to get more insight into the kinetics of oleandrin excretion in dairy milk, particularly concerning its depletion after cow recovery and/or after the removal of the contaminated feed. This would help both in protecting consumer health and in reducing the negative economic impact for the farmers due to milk discarding.

## 4. Materials and Methods

### 4.1. Animals

The study was approved on 21 June 2018 by the Ethics Committee for Animal Experimentation, of the Department of Veterinary Medicine, University of Bari (Prot. No. 12/18).

### 4.2. Clinical Investigations

Complete blood count (CBC) was performed using the Abbott cell counter analyzer CELL DYN 3700 (Abbott, Chicago, IL, USA). Serum biochemical profile (aspartate transaminase—AST-, alanine transaminase—ALT-, ALP, CK, LDH, total protein, albumin, cholesterol, non-esterified fatty acids, urea, creatinine, glucose, calcium, phosphorus, magnesium, sodium, potassium, chloride, and iron) was assessed with the LYASIS photometer (Assel, Guidonia-Roma, Italy)with interferential filters. ECG was performed with a portable electrocardiograph (Esaote P8000, Esaote, Genova, Italy).

### 4.3. Necropsy and Histopathological Analysis

Necropsy was performed on all the dead subjects according to standard procedures, and the following organs were collected: heart, liver, kidney, spleen, esophagus, small intestine, large intestine (colon and cecum), and mesenteric lymph nodes. Different cardiac areas were sampled, namely, interventricular septum, left papillary muscle, right papillary muscle, left ventricular wall, right ventricular wall, left atrial wall, right atrial wall, mitral valve, and tricuspid valve. The samples were fixed in 10% buffered formalin solution, routinely embedded in paraffin wax blocks, sectioned at a 5-μm thickness, mounted on glass slides, and stained with Haematoxylin and Eosin for histopathological examination.

### 4.4. Toxicological Analysis

Blood, heart, and liver samples from three animals were collected and processed as reported in Gosetti et al. (2019) [33]. A milk sample from three poisoned cows and a sample of cheese produced with suspected contaminated milk were collected and stored at −20 °C until the analysis.

For oleandrin extraction and purification from milk and cheese, 4 g of cheese or 15.0 mL of milk, were accurately weighed and put in a 50 mL tube, in which 2.0 mL of H_2_O/methanol (MeOH) 50/50 (*v*/*v*) was added, and homogenized with vortex for 1 min. Then, 21.0 mL of acetonitrile and 4.0 mL of acetone were added; the sample was homogenized with Ultraturrax (VWR, Werke, Staufen, Germany) at 15,100× *g* for 1 min and successively centrifuged at 1500× *g* for 10 min at 4 °C. An aliquot of 30 mL of the resulting clear extract was evaporated under nitrogen at room temperature and dissolved with 1.0 mL of hexane (the lipid fraction layer at the bottom of the tube) ready for solid-phase extraction (SPE) treatment.

The SPE was carried out using the cartridge Strata FL-PR Florisil (1 g, 6 mL) by Phenomenex (Milano, Italy). The cartridge was conditioned with 10.0 mL of hexane and loaded with 1.0 mL of sample extract. The washing step was performed by 10.0 mL of ethyl acetate/hexane 5/95 (*v*/*v*). The cartridge was dried under vacuum for 2 min and then eluted with 15.0 mL of ethyl acetate. The eluted solution was evaporated under a gentle stream of nitrogen and dissolved in 250.0 μL of MeOH, and the extract was filtered on a 0.2 mm PTFE filter (VWR, Darmstadt, Germany).

The detection of oleandrin in tissues, biological liquids, and cheese was performed by an UHPLC-MS/MS analysis as previously reported [33]. A method validated for bovine blood, serum, heart, and liver was tested for milk and cheese samples, and validated according to International Council for Harmonization of Technical Requirements for Pharmaceuticals for Human Use (ICH) guidelines. The method detection limit (MDL) and the method quantification limit (MQL) values obtained for milk were 0.018 and 0.055 ng mL^−1^, respectively, whereas those for cheese were 0.010 and 0.029 ng g^−1^, respectively. No evidence of matrix effect was found for both matrices. The recovery (R) was evaluated at three concentrations (1.0, 250, and 500 ng mL^−1^) and the obtained R values at the different concentrations were not statistically significant at 95% confidence level, indicating that R values do not depend on the analyte concentration in the explored concentration range. The average recovery values obtained for milk and cheese samples were 74.8 ± 5.8 and 70.5 ± 4.9, respectively.
